# The Effect of *Zizyphus Jujube* Fruit Lotion on Breast Fissure in Breastfeeding Women

**Published:** 2018

**Authors:** Nasim Shahrahmani, Sedigheh Amir Ali Akbari, Faraz Mojab, Moghadameh Mirzai, Hadis Shahrahmani

**Affiliations:** a *Student Research Committee,Department of Midwifery, School of Nursing and Midwifery, Shahid Beheshti University of Medical Sciences, Tehran, Iran. *; b *Department of Midwifery, School of Nursing and Midwifery,Shahid Beheshti University of Medical Sciences, Tehran, Iran. *; c *Department of Pharmacognosy, Shahid Beheshti University of Medical Sciences, Tehran, Iran. *; d *Public Health School, Kerman University of Medical Science, Kerman, Iran.*

**Keywords:** Breastfeeding, Breast fissure, *Zizyphus jujube*, Pain, Complementary medicine

## Abstract

Nipple fissure is a common problem during breastfeeding. *Zizyphus Jujube* Fruits is one of the oldest medicinal plants which can heal wounds through its antimicrobial and anti-inflammatory properties. This study aimed to determine the effect of *jujube* lotion on the recovery of breast fissure. This double-blind clinical trial recruited 100 primiparous lactating women who were randomly divided into two groups. In Jujube group, mothers used 0.5 mL of Fruit Lotion, and in control group mothers applied 4-5 drops of their breast milk five times a day, after breastfeeding. Both groups were examined on the 7th and 14th days after childbirth. The damage severity was assessed using the Amir scale and the presence or absence of nipple discharge was recorded. A significant difference was observed between the two groups in the extent of nipple damage before intervention on the 3^rd^ day after childbirth and after intervention on the 7^th^ and 14th days after childbirth *(P* = 0/02، *P* = 0/000). No significant difference was observed in sore nipple discharge between the two groups before the study and on the 7th day, while a statistically significant difference was observed between the two groups on the 14th day (*P* = 0/1, *P* = 0/01). The finding of this study revealed that the *Zizyphus jujube* fruits lotion heals nipple fissure faster and better than breast milk.

## Introduction

Breast milk is the gold standard and the safest way to feed infants in the world (1, 2). After childbirth, mothers may experience discomfort, mild pain, and sore nipples in the first breastfeeding. Nipple fissure or sore nipple is a macroscopic lesion observed on the nipple and areola of lactating mothers as cracks, destruction of skin, wounds, and clinical signs of erythema, edema and blistering (3). The prevalence of nipple fissure has been reported between 34% and 96 % (4, 5 and 6). Unbearable pain caused by nipple fissure leads to mothers’ emotional distress, disruption in mother-baby relationship, and early termination of breastfeeding. Cracked nipples create a point of entry for bacteria which can cause mastitis and abscesses (4, 7). Therefore, when nipples are stimulated and cracked, therapeutic measures should be taken for healing wounds, relieving pain, reducing healing time for successful breastfeeding, exclusive breast feeding, and preventing infection (3, 6 and 7). Moist wound healing has been known as the most effective way for reducing tissue destruction and increasing epithelialization (8). Breast milk is a simple, available, secure, and free treatment and it also reduces the period of nipple pain. Since breast milk has different kinds of antibodies, anti-inflammatory and anti-bacterial features, it is really recommended for treatment of nipple pain (5, 9 and 10). But several studies have shown that in some recommended treatments for improvement of nipple pain, there is no superiority between using hot water compresses, tea bag compresses, lanolin, breast milk, glycerol pad, collagenase, vitamin A, honey, and lanolin versus breast protector (10, 16 and 11). 

In general, flavonoid and phenolic compounds that herbal extracts produce help neutralize free radicals or exert their biological effects, so they are usually used for the treatment of topical wounds (12, 13). 

*Zizyphus jujube* Fruit is one of the species used for the treatment of wounds and burns. The *Zizyphus jujube* Fruit has been described as the fruit of life. Ancient Chinese discovered the unique and medicinal properties of this fruit thousands of years ago (14, 15).

The *Zizyphus jujube* Fruit, scientifically known as *Ziziphus jujube* Miller, belongs to *Rhanmaceae* family. *Zizyphus jujube* fruit contains fatty acids, beta-Carotene, Alpha-Tocopherol, 7 phenolic compounds, caffeic acid, epicatechin, ferulic acid, routine, p-hydroxybenzoic acid, chlorogenic acid, vitamin A, C, E, ferulic acid, flavonoids, cyclopeptide, and tanan. *Zizyphus jujube* Fruit exerts its beneficial effects on tissues with its anti-inflammatory, anti-allergic, antiseptic, antiviral, antioxidant, antifungal, and bactericidal properties (13, 14, 15, 16, 17, 18, 19 and 20).

Rafeeian *et al.* (1389) have studied the healing effect of *Zizyphus jujube* lotion on burn injuries of mice and showed that the *Zizyphus jujube* lotion is effective for healing those wounds (14). Setorki (2016) , in a study about anti-inflammatory activity and anti-oxidant feature of* Zizyphus jujube* lotion on mice, came to this conclusion that this fruit is effective for lowering inflammatory in ulcerative colitis and these effects all happen due to the existence of some compounds such as gallic acid, catechin, caffeic acid, coumarin a chlorogenic acid in the fruit (21).

Ping *et al.* (2000) studied Analgesic, anti-stress, and sedation effects of *Zizyphus jujube* juice in an experimental environment on mice and the results indicated that *Zizyphus jujube* juice has analgesic, anti-stress, and sedation effects and the researcher believed that these effects happen due to decrease in the activities of monoamines (22).

Moreover, its effect on the collagen content and epithelialization of tissues and acceleration of wound healing has been proved. Given the importance of breastfeeding and high prevalence of nipple fissure and its effects on the early termination of breastfeeding, this study was conducted to determine the effect of *Zizyphus Jujube* Fruit lotion on the recovery of breast fissure due to its unique properties and effects on wound healing.

## Experimental

This double-blind clinical trial was registered in the Iranian clinical trial database (IRCT2015121225490N1) after being approved by the ethics committee. This study recruited primiparous women presenting to the Obstetrics and Gynecology Clinic of Ghaem Hospital affiliated to Kerman University of Medical Sciences on the third day after childbirth for screening their newborns for hypothyroidism (a routine screening in Iran) from October 2015 to March 2016 and they were examined in terms of breast fissure.

Written informed consent was first obtained from mothers after explaining the purpose of the study and the methodology. 

The samples which had entrance criterion features were selected and then they were randomly placed in two groups of breast milk and *Zizyphus jujube* lotion by using excels software. In this study, after consulting with a professor in field of statistics, the numbers of samples were achieved by the following relation: 

The least required number of samples in the following relation is 50 persons.

n=Z1-α22p¯1-p¯2+Z1-βp11-p1+p21-p22p1-p22

α=0.05          →z=1.96

$p_{1}$=0.90          $p_{2}$=0.70

$n_{1}$=$n_{2}$=44          $\bar{p}$ =$\frac{P_{1}+P_{2}}{2}$

𝛽 = 0. 2 →z=0.84

P_1_ is the percentage of recovery in *Zizyphus jujube* lotion group and P_2_ is the percentage of recovery in breast milk group (32). Based on this formula, at least 44 samples were estimated for each group. By considering a 10% to 15% possibility of decreasing the number of samples in different levels, 50 persons were estimated for each group and 100 samples were estimated for this study overall (33).

Inclusion criteria were Iranian nationality, literacy, having a singleton pregnancy, being primiparous, mothers having no breast abnormalities (flat or inverted nipples or any clear deformation of the nipple due to surgery or burns), baby’s birth weight between 2500 and 4000 gr., baby’s not having frenulum breve, baby’s not having minor anomalies of the mouth, palate, jaw, and face, lack of allergy to topical medications, exclusive breastfeeding, non-use of an artificial nipple and breast pump, mothers’ not having diabetes or any other internal diseases and mental disorders.

Exclusion criteria were unwillingness to continue participating in the study, the use of pacifiers, artificial nipples, breast pumps, and milk powder during the study, being allergic to *Zizyphus Jujube* Fruits lotion, using medications for sore nipples or analgesics during the study, using suggested treatments fewer than four times, having puerperal fever, mastitis, and baby’s developing oral infections and diseases. In this study, the data were collected through demographics and obstetrics questionnaire, health assessment form, visual analogue scale, Amir Scale, nipple discharge scale, and inventory *of Zizyphus jujube Fruit* lotion side effects. Content validity was used to determine the validity of the demographics and obstetrics questionnaire, health assessment form, and inventory of *Zizyphus jujube* Fruit lotion side effects (including: sensitiveness, infection, itching, smart). To determine the reliability of the health assessment form, the test re-test was used on the third and 14th days and its reliability coefficient was obtained 0.95. The Amir scale was used to evaluate nipple fissure. In this scale, zero means no damage, 1-2 mm mild damage, 3-9 mm moderate damage, and 10 mm or higher severe damage. This scale is a valid instrument, whose reliability and validity have been confirmed in the previous studies (25, 26). The inter-rater reliability test was used to determine the reliability of the Amir scale. Accordingly, the nipple fissure recovery form was simultaneously completed for 10 participants by the researcher and one of the colleagues on the 7th day after childbirth and the reliability coefficient was obtained 0.85 indicating that this scale is reliable. 

Study units, in case they match the entrance criterion, enter this study as samples and then by using excel software (Ran between function) they divide randomly into two 50-person groups of breast milk and *Zizyphus jujube* lotion, and afterward the samples were placed in determined groups based on the provided list from the order of samples entrance to this study. 

*Zizyphus jujube* lotion was made as following: first, 700 grams of fresh *Zizyphus jujube* fruit was bought from the traditional bazar of Kerman, the pits were removed after it was dried, and the rest of it was powdered. 210 mg of the juice of *Zizyphus jujube* fruit with 54% efficiency was made by using 96%-ethanol by pharmacy department of Shahid Beheshti University. 

At the day of referral, before treatment, correct techniques of breastfeeding were instructed face to face and the benefits of breastfeeding were explained to mothers for mothers’ and babies’ health (in order to encourage mothers to continue breastfeeding and their treatment). Mothers feed their infants in front of the researcher and correct method of breastfeeding was evaluated and Amir Scale was completed.

Considering the similar studies, in order to coordinate the receiving amount and usage times of breast milk and *Zizyphus jujube* fruit lotion, the following method was applied:

Women in the *Zizyphus Jujube* Fruit lotion group were advised to apply 0.5 mL of *Zizyphus jujube* Fruit lotion on the nipple and areola 5 times a day after each breastfeeding, and women in the breast milk group were advised to apply 4-5 drops of their own milk on the nipple and areola 5 times a day after each nursing. The women in both groups were asked to go to the relevant hospital on the 7th and 14th days after childbirth to assess extent of damage and presence or absence of discharge from sore nipples (25, 26 and 27). 

The extent of damage and presence or absence of nipple discharge was recorded by an assistant researcher blinded to the type of treatment. If patients refused to attend the hospital, the assistant researcher examined their breast fissures by going to their homes.

Also to analyze the data in this research, a researcher-aid proficient in statistics helped us without letting her know the treatment type, and SPSS software (version 22) was used.

Given that the response variable was ordinal and the data were not normally distributed, extent of damage were first compared to three days using Friedman test and then, the responses were compared between the two groups using the Mann-Whitney test. Furthermore, in cases in which Mann-Whitney test was statistically significant, therapeutic cycles were compared mutually using the Wilcoxon test. To examine the presence or absence of discharge from sore nipples, a comparison was made using the Cochran test in three days and responses were then compared between the two groups using the chi-square test.

The two groups did not have any sample fall and also no side effect of using *Zizyphus jujube* lotion was reported on the side of mother.

## Results

This study recruited 100 primiparous lactating women with sore nipples who were divided into two groups of 50 receiving *Zizyphus jujube* Fruit lotion and breast milk. Obstetrics and demographics characteristics are presented in (Table 1). Both groups were matched for level of education, occupation, age, income, baby’s birth weight, gestational age, mode of delivery, and health status.

There was no significant difference in the mean nipple damage between the two groups before the study (*p* = 0.8). There was a statistically significant difference in lesion severity score on the third day after childbirth (before intervention) and the 7th day after childbirth (after intervention), as well as on the third and 14th days after childbirth between the two groups (Table 2). There was a significant difference in the presence of nipple discharge on the third day before intervention and the 7th day after birth and the third day before intervention and 14th day after birth between the two groups. There was no significant difference in the nipple discharge between the two groups on the 7th day after childbirth (Table 3).

## Discussion

The results showed that *Zizyphus jujube* Fruit lotion heals sore nipples faster than breast milk. A significant reduction was observed in the mean nipple damage, pain severity, and nipple discharge on the 7th and 14th days after treatment with *Zizyphus jujube* Fruit lotion compared with the use of breast milk, indicating that sore nipples healed faster in the group receiving *Zizyphus jujube* Fruit lotion than in the breast milk group. The pain severity and nipple discharge in the *Zizyphus jujube* Fruit lotion group were less than those in the breast milk group. In fact, wound healing is a complex process: infiltration of inflammatory cell occurs in the early days, and reduces on the second and third days when fibroblast proliferation, collagen synthesis, and angiogenesis occur. Also, wound contraction occurs throughout the wound healing process. Numerous studies have proven that specific compounds in some plants are effective for healing wounds and reducing discomfort in the shortest time possible (17, 28). Rafieian, in their study on the effect of hydro-alcoholic extract of *Zizyphus jujube* Fruit ointment on burn wound healing process in mice, reported that burn wound healed more in the group receiving jujube ointment 1% compared with control group and groups receiving Vaseline and jujube extract ointment 10% (*P* < 0/001, *P* < 0/05, *P* < 0/05). In this study, jujube ointments 1% and 10% were used to evaluate burn wound healing, which revealed that jujube ointment 1% was more effective than jujube ointment 10%. Therefore, by increasing concentrations of jujube extract, the wound healing effect reduced. This study suggested that the healing effect of jujube is due to its large amounts of unsaturated fatty acids, flavonoids, and tryptonoids. Fatty acids increase collagen synthesis and ascorbic acid, through decreasing macrophages, increasing fibroblasts, stimulating angiogenesis, and anti-inflammatory effects improving healing wounds (14, 29).

In the present study, existence of flavonoids and steroids in *Zizyphus jujube* can lead to reduction of nipple fissure inflammation and its curing. Also due to its anti-allergy effects, allergies were not seen during *Zizyphus jujube* lotion treatment; the results of this study match our research.

As reported in Goyal’s study, with increasing concentrations of jujube extract, its anti-inflammatory effect (by reducing the expression of nitric oxide) increases. 200 and 400 mg/Kg of jujube extracts can reduce acute and chronic inflammation caused by carrageenan by reducing the expression of nitric oxide and prostaglandins (*P* < 0/05). At doses of 400 and 200 mg/Kg, jujube extracts reduced inflammation 47.4% and 35.5%, respectively. In this study, alkaloids, flavonoids, glycosides, and terpenoids were found in the jujube juice. Flavonoids and terpenoids have anti-inflammatory and anti-allergic properties by reducing the expression of nitric oxide, prostaglandins, and reducing histamine release (18, 19 and 20).

**Table 1.** Comparison of demographic and midwifery characteristics of *Zizyphus jujube* Fruit and breast milk groups in primiparous lactating women

**variable**		***Zizyphus jujube*** ** Fruit** **(n = 50)**	**Breast Milk** **(n = 50)**	***P*** ** -value**
Age (Years)	3.43±24.16		3.51±23.18	P=0.1*
Having Job (House Wife)	(62)31		30(60)	P=0.8**
Education(High School)	(38)19		(40)20	P=0.9**
Gestation Age(38 Weeks)	1.06±38.14		0.99±38.10	P=0.8*
Delivery Mode (NVD)	(68)34		(64)32	P=0.8**
Baby’s Birth Weight(Gr)	325.92±3113.18		370.77±3170.00	P=0.3*
Health Status (Average)	(58)29		(62)31	P=0.7**
Income family (Average)	(48)24		(52)26	P=0.9**

The result are presented as mean (SD) or number(%): SD = standard deviation

* Two Independent sample T Test

** Chi-Square Test.

**Table 2:** Comparison of Nipple fissure damage severity before and after treatment in *Zizyphus jujube *Fruit and breast milk groups in primiparous lactating women

**group**	**Before intervention** **(n = 50)**	**4 day after intervention** **(n = 50)**	**11 day after** **Intervention** **(n = 50)**	***P*** ** value** *
Zizyphus Jujuba Fruit	2.02 ± 0.65	1.24 ± 0.85	0.68 ± 0.52	p < 0.001
Breast milk	0.68 ± 2.16	0.88 ± 1.58	1.03 ± 1.16	P < 0.004
P value**	P = 0.8	P = 0.02	p < 0.001	

* Friedman

** Mann- Whitney

*** Millimeter, The result are presented as Mean ± SD, SD = Standard Deviation.

**Table 3.** Comparison of Nipple fissure discharge before and after treatment in *Zizyphus jujube* Fruit and breast milk groups (mean ± SD) in primiparous lactating women

**group**	**Before intervention** **(n = 50)**	**4 day after intervention** **(n = 50)**	**11 day after** **intervention**	***P*** ** value** *
Zizyphus Jujuba Fruit%	68	40	22	p < 0.001
Breast milk%	64	54	44	P < 0.01
P value**	P = 0.6	P = 0.1	p < 0.001	

* cochran

** chi- square, SD = Standard deviation

**Table 4:** Wilcoxon test to compare both groups Barge

**Comparison**	**P value**	**Compare injury recovery**
A,B	*P<0/001*	B>A
A,C	*P<0/001*	C>A
B,C	*P<0/004*	B<C
D,E	*P<0/001*	D<E
D,F	*P<0/001*	F>D
E,F	*P<0/001*	F>E

A: Zizyphus jujube Fruit lotion before intervention

B:Zizyphus jujube Fruit lotion 7th day after childbirth (after intervention)

C: Zizyphus jujube Fruit lotion 14th day after childbirth (after intervention)

D: breast milk before intervention

E: breast milk 7th day after childbirth (after intervention)

F: breast milk 14th day after childbirth (after intervention

**Figure 1:**
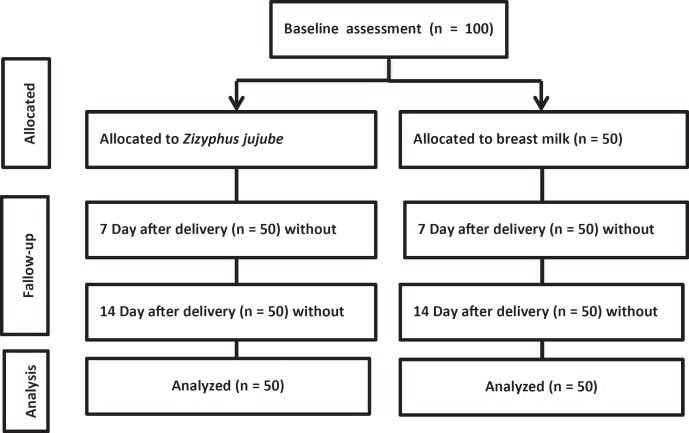
Flowchart of the study

The results of this study match ours due to large amount of these compounds in *Zizyphus jujube*. Anti- fissure and anti-inflammatory effects of *Zizyphus jujube* can be explained through this study.

A study conducted on the effect of jujube peel ointment on wounds in mice found that wounds healed more quickly in the group receiving *jujube* peel ointment 10% W/W compared with the control group and the group receiving *jujube* peel ointment 5% W/W, which was comparable to the group using betadine in this study (*P* < 0/001). The wound healing potential of *jujube* peel extract has been attributed to its tannin and flavonoid compounds which cause wound contraction and increase protein content and epithelialization of the wound (17). Thus, the presence of these compounds in *jujube* with similar effects is consistent with the results of our study. According to Kumar’s study, doses of 200, 400, and 600 (mg) of *jujube* peel extract and 100 (mg/Kg) of diclofenac sodium had inhibitory effects on inflammation. This inhibitory effect at a dose of 600 mg of *jujube* peel extract was higher than other doses, but less than the diclofenac sodium group (29).

Due to the large amounts of polyphenol, steroid, vitamin C and A in the skin of *Zizyphus jujube*, we can explain anti-inflammatory effects of this fruit. In the study done on the extract of *Zizyphus jujube* leaf in reducing inflammation, it was seen that the leaf of *Zizyphus jujube* has compounds such as phenols, terpenoids and epi catechin, which avoid the spread of inflammatory mediators (30). On the other hand, epi catechin, as an antagonist which receives serotonin and cyclopeptide, acts as a controller of calmodulin both of which have anti-pain effects. Therefore, by reducing nipple pain, discharge of inflammatory mediators will be avoided (31). The results of both researches match our study due to large amounts of these compounds in *Zizyphus jujube* and anti-inflammatory effects of *Zizyphus jujube* can be explained through this study.

In other similar studies, olive oil and green tea with compounds similar to jujube fruit were effective for the recovery of breast fissure (32, 33).

In our study, the *Zizyphus Jujube* Fruit lotion was effective in reducing nipple pain due to having compounds, such as cyclopeptides, catechins, and phenolic acids (34, 35). According to the results of this study and other similar studies, the jujube fruit seems to be effective in the treatment of breast fissure due to having compounds, such as saponins, fatty acids, flavonoids, terpenoids, phenolic compounds, Beta-carotene, vitamins A and C, and having antioxidant, anti-inflammatory, antimicrobial, antifungal, and anti-ulcerogenic properties (36, 37, 38, 39 and 40). 

## Conclusion

The results showed that Zizyphus Jujube Fruit lotion can treat sore nipples faster than breast milk over a period of 10 days. Also, nipple pain in the jujube lotion group was less than the breast milk group. Given the effect of jujube lotion on the recovery of breast fissure and lack of adverse side effects in this study, it seems that the Zizyphu Jujube Fruits lotion can be used for the treatment of breast fissure. 
